# ChaQra: a cellular unit of the Indian quantum network

**DOI:** 10.1038/s41598-024-67495-8

**Published:** 2024-07-20

**Authors:** Shashank Gupta, Iteash Agarwal, Vijayalaxmi Mogiligidda, Rajesh Kumar Krishnan, Sruthi Chennuri, Deepika Aggarwal, Anwesha Hoodati, Sheroy Cooper, Ranjan, Mohammad Bilal Sheik, K. M. Bhavya, Manasa Hegde, M. Naveen Krishna, Amit Kumar Chauhan, Mallikarjun Korrapati, Sumit Singh, J. B. Singh, Sunil Sud, Sunil Gupta, Sidhartha Pant, Sankar, Neha Agrawal, Ashish Ranjan, Piyush Mohapatra, T. Roopak, Arsh Ahmad, M. Nanjunda, Dilip Singh

**Affiliations:** 1 QuNu Labs Pvt. Ltd., M. G. Road, Bengaluru 560025 Karnataka, India; 2 Okinawa Institute of Science and Technology Graduate University, Okinawa, Japan

**Keywords:** Optics and photonics, Physics

## Abstract

Major research interests on quantum key distribution (QKD) are primarily focused on increasing 1. Point-to-point transmission distance (1000 km). 2. Secure key rate (Mbps). 3. Security of quantum layer (device-independence). It is great to push the boundaries in these fronts but these isolated approaches are neither scalable nor cost-effective due to requirements of specialised hardware and different infrastructure. Current and future QKD network requires addressing different set of challenges apart from distance, key rate and quantum security. In this regard, we present ChaQra—a sub quantum network with core features as 1. Crypto agility (integration in the already deployed telecommunication fibres). 2. Software defined networking (SDN paradigm for routing different nodes). 3. reliability (addressing denial-of-service with hybrid quantum safe cryptography). 4. upgradability (modules upgradation based on scientific and technological advancements). 5. Beyond QKD (using QKD network for distributed computing, multi-party computation etc). Our results demonstrate a clear path to create and accelerate quantum secure Indian subcontinent under national quantum mission.

## Introduction

Securing critical infrastructure is most critical at present due to continuing advancement in classical super computing and quantum computing. Referring to the Mosca’s Inequality^1^, this threat is not just a future concern but a present one. Quantum problems have quantum solutions. To address this quantum threat, there are two well-known strategies that are being explored at present. 1. Quantum Cryptography: Most promising approach based on the fundamental laws of quantum mechanics. All classical information processing tasks and beyond (quantum teleportation and super dense coding) can be done securely^2–5^. Quantum key distribution (QKD) is the most advanced and well developed under this strategy with the objective of deriving symmetric keys between the communicating parties via insecure public channel with the unique ability to detect the eavesdropping. Quantum Secure Direct Communication (QSDC) is another avenue of the Quantum Cryptography where secure communication is possible without the requirement of the shared keys^6–14^. 2. Post-Quantum Cryptography^15^ which uses the conventional cryptography to develop alternative public key encryption schemes that are hard even for quantum computer to break. However, these are secure against the known quantum attacks whereas theoretical security proofs of QKD protocols is independent of all future advances in computational power or algorithms. This field has seen significant progress in recent years, with rigorous standardization processes at NIST and ISO, and the first selection of NIST standards expected to be available in 2024. In the noisy intermediate era, hybrid approach is the recommendation of the standard bodies and cybersecurity professionals to address the challenges and drawbacks of both approaches. Several demonstrations of QKD and QSDC^16–20^ not just in a laboratory but real world environment have been achieved over the years Table 1. However, large scale secure quantum network connecting any two given points across the globe is still far from reality.

In point-to-point (P2P) QKD, recent advancements in twin-field QKD^21^ have broken the fundamental repeaterless secret key capacity bound (SKC$_0$)^22,23^. There is an intermediate untrusted quantum relay node between the end users to give secret capacity beyond SKC$_0$. However, it works only when the phase and frequency of the independent photon sources at the end users matches which is extremely hard and unsustainable in field environment. Decoy based schemes are better, stable and efficient in field environment with secret key rate scaling linearly with the loss^24–26^. In the long term, (semi-) device-independent QKD^27^ with the ability to demonstrate loop-hole free Bell-test^28^ (quantumness verification) at telecom wavelength might be a promising approach with scalability (using quantum repeaters) and selftestability.

Point-to-Multipoint (P2M) is the first step towards quantum communication network^29^ by scaling the standard two-user QKD configuration to many users. QKD networks can be categorised as 1. Trusted node networks: It offers quantum secure links with best classical or post-quantum secure intermediate node^30–33^. 2. Actively switched networks: It uses time or wavelength division multiplexing to connect certain pair of users to derive secure keys at a given time^34–37^. 3. Fully connected quantum networks: It is based on high dimensional or multipartite entanglement to generate secure keys between any or all the users in the network^38,39^. Most of the field trials or quantum network demonstrations falls under category-1 or 2. These demonstrations followed an agile approach where secure key distribution is achieved on deployed fibres with high bandwidth data traffic. Such agile approach at present yields polynomial cost reduction by time-multiplexed Hub and Spoke configurations. Network resilience against link disruption is mitigated using multiple QKD links between end users. This might not be the case during the initial QKD network deployment. Therefore, different automated hybrid mechanism should be there to address the link disruption for continued network operation.

The primary objective of the QKD networks is secure key distribution. However, their utility is much more in the quantum era. One such application is in the field of distributed computing with specific use case in the secure multiparty computation^40^. The shared randomness in the QKD network can be used to solve problems like private set intersection or aggregation where the key idea is to compute a function jointly while keeping respective party inputs private. Currently this kind of multiparty computation is done using public key cryptography which will not be secure in the post-quantum era. Thus, the usage of QKD networks extends far beyond just key distribution. This worthiness of the QKD network together with the modular hardware approach is becoming increasingly popular in the development of commercial Quantum Key Distribution (QKD) products. Such flexibility in the design and implementation of QKD systems is absolutely necessary due to continuing advancements being made both at the source node and detector node components. Such developments can be incorporated into the deployed systems without the need for complete system overhauls. For instance, improvements in the efficiency of single-photon detectors or the stability of quantum light sources can be integrated as individual modules. Furthermore, it facilitates interoperability between different QKD systems and promotes the development of standardized components, both of which are crucial for the widespread adoption of quantum communication technologies. We keep this approach at our core for developing the Armos QKD nodes^41^ and simulators^42,43^.

In this paper, we present a cellular unit of the Indian quantum network mentioned as ChaQra (derived from the Sanskrit word ‘Chakra’ meaning a ‘disk’ or ‘wheel’ symbolising Hub and Spoke topography of the QKD network) afterwards. It is designed and implemented by the QuNu Labs Pvt. Ltd. (an Indian start up incubated from Indian Institute of Technology, Madras) with the support of the Indian defence force. The paper summarizes the point-to-multipoint (P2M) approach to QKD networks with a focus on software-defined networking (SDN) for switching between different spokes and the trusted repeater paradigm. It is a hybrid of the category-1 (trusted node) and 2 (actively switched) of the QKD networks. It discusses the architecture and functionality of the ChaQra key generation, key management and key migration layer. We demonstrate the round-robin and on-demand switching mechanism between a single centralised node (Hub) and five branch nodes (spokes). We further demonstrate a secure (AES-256 encryption seeded with QKD keys) video-conference (polycom) with all deployed nodes even under the denial-of-service situation using a custom Q-Infinity module highlighting basic mechanisms of the continuous and reliable operation of the ChaQra network.

The paper gives an overview of a single point-to-multipoint network link and their underlying key generation, key management and key migration technology: five QKD transmitter units (spokes) are connected to a single QKD receiver unit (Hub) based on time-division multiplexing (TDM) signifying polynomial reduction in cost with respect to the point-to-point QKD. Depending upon the link loss, key generation protocol is either Differential phase shift (DPS) upto 20 dB or Decoy-based DPS (upto 33 dB). The paper also covers the experimental simulation of the ChaQra using Matlab and Simulink and establishes a link between the simulated and observed results.

The paper is organised in the following way. In “Recapitulate”, we recapitulate the major QKD networks across globe with focus on their key features and milestones achieved. In “Hub and spoke topology (ChaQra)”, we provide the network diagram, architecture, SDN functionalities, and network extension of ChaQra while “QKD systems” provide the two kind of QKD systems considered. In “ChaQra: experimental simulation”, we explain the Simulink blocks (Hub, Spoke, and optical switch) of the experimental simulation and “Methods”, elaborates the constituting layers of the ChaQra sub-network. Finally, we conclude with a discussion and summary of our results in “Discussion”.

## Recapitulate

In this section, we briefly discusses some previously deployed QKD networks and its unique features (Table 1). Since much of interests include the literature that deals with quantum key distribution in the existing optical infrastructure, distance, and security, the focus is placed on the logical structure of networks and topology, key storage and management solutions, key usage, and the solution’s performance.

**Table 1:** Quantum key distribution network across world.

S.no.	QKD-network	Key features	References
1.	USA DARPA QKD network and *Phio network* by Quantum Xchange (USA)	The first QKD network demonstration (2002). Switching mechanism is based on Dijkstra’s algorithm (Bae or cross connection) and trusted node capabilities. Proposed QKD link between Washington DC and Newyork city. *Protocol:* BB84; BBN protocol suite; modified IPSec and OSPFv2; QPFS. *Max key rate:* 400 bps at Max P2P distance of 29 km.	^36,44–46^
2.	EU SEOCQC QKD network	Integration of both prepare and measure and entanglement based QKD (2004). Hop by hop networking and long term uninterrupted operation (six months). *Protocol:* BB84 and BBM92; Q3P protocol suite; extension of OSPFv2 routing. *Max key rate:* 3.1 kbps; Max P2P distance of 82 km.	^30,47^
3.	Japan Tokyo UQCC network	Hierarchical management and key management servers for six nodes (2010). *Protocol:* BB84 and BBM92. *Max key rate:* 3.1 kbps; Max P2P distance of 33 km.	^48,49^
4.	China (Hefei, Chaohu, and Wuhu) network	150 km of coverage area and 5000 h operation (2014–2021). 14 QKD devices over 11 nodes. Extended to 46-node quantum metropolitan-area network. *Protocol:* Phase coding based decoy state BB84. Client-server architecture to maximize channel utilisation.	^31,33^
5.	China (Wuhan metropolitan) QKD network	Centralised command and control centre (2017). 26 trusted repeater nodes and 95 end user nodes (82 QKD links). Network build by Quantum CTek. WDM technique (service+quantum+classical). IPSec VPN protocol seeded with QKD keys.	^50^
6.	China (Jinan) QKD network	8000 km$^2$ area covered with dark fibre and 437 end user nodes (2017-21). Build by Quantum CTek and China union Shandong branch. *Protocol:* polarisation encoding based decoy state BB84; IPSec VPN with refresh rate of 1 second.	^51^
7.	Cambridge quantum network	P2M point (3 nodes) Metropolitan QKD network in already deployed fibre. Quantum Link disruption is addressed by link redundancy. 10k+ user support via quantum access networks (QANs). *Protocol:* Phase encoded BB84 with decoy states continuously operated over 580 days with key rate > 2 Mbps.	^52^
8.	ChaQra-Indian Quantum Network	Hybrid approach to address denial-of-service. SDN support with three-factor authentication and tamper-proofing. WDM technique for integration with already deployed fibre. *Protocol:* Decoy based DPS. *key rate:* 1 kbps at Max P2P distance of 150 km.	This work

Apart from these popular QKD network, there have been recent advancements in the coexistence effort of the QKD and optical transport network on the deployed fiber with data channels operating at high launch power^53–57^. Addressing practical challenges to in QKD network^58^, advancements in integrated quantum communications^59^, QKD with on-chip light sources^60^, cost efficient quantum networks^61,62^, and Hybrid QKD network^63–68^.

The uniqueness of the ChaQra stems right from 1. The protocol selection to address photon number splitting attacks along with longer secure links and higher key rate. 2. Multifactor Authentication comprises factory paired keys flushed after first round of secure key generation together with post quantum cryptographic authentication. 3. Tackling denial-of-service attacks using Q-infinity (PQC enabled quantum safe cryptography). 4. Coexistence with the data channel in already deployed fiber network using wavelength division multiplexing and spectral filtering. 5. AI enabled network control using Software-Defined Networking (SDN). 6. Cost-effective topography with single receiver node and multiple transmitter node to cater multiple users in a quantum access network. 7. Beyond QKD approach to leverage the QKD network for quantum secure multiparty computation and distributed computing. There are several unique features like multiprotocol support, on-chip implementation of the spokes, transceiver design are in progress. Next, we discuss the topography and architecture of ChaQra.

## Hub and spoke topology (ChaQra)

**Figure 1:**
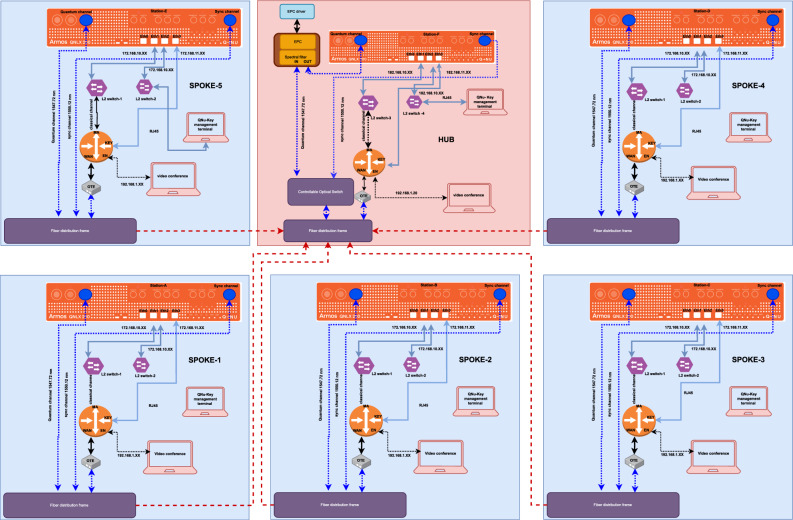
Network diagram of a Hub with five spokes. Note that the switching mechanism is a part of the central control station (Hub). Key management terminal is accessible through L2 switch-2. Key migration to multiple application takes place via L2 switch-1. All the stages for key generation starting from authentication, quantum state preparation (spokes), quantum state measurement (Hub), sifting, parameter estimation, error-correction, privacy amplification, key verification occurs inside the 19 inch box shown as orange rectangular box. Dotted blue lines shows service channel (DWDM channel number 34) and quantum channel (DWDM channel number 37). The transmission length between the Hub and spoke-A1, A2, A3, A4, and A5 are 100 km (28 dB), 100 km (26 dB), 75 km (18 dB), 65 km (15 dB), and 100 km (30 dB) respectively.

The ChaQra sub quantum Network consists of a single centralized network monitoring and detection unit (Hub) and five transmitter unit (extendable) mentioned as spokes in Fig. 1. Hub is capable of tuning and aligning the optical signals with the spokes on startup and store these tuning parameters for further use (reducing the switching time in the next rounds). The spokes are connected by a bundle of deployed telecommunication fibers of varying attenuation strength (0.2dB to 0.36 dB). These fibers include many splicing points and connectors causing the links to be quite lossy. The transmission length between the Hub and spoke-A1, A2, A3, A4, and A5 are 100 km (28 dB), 100 km (26 dB), 75 km (18 dB), 65 km (15 dB), and 100 km (30 dB) respectively. The DWDM channel number-34 is used for the service link and 37 is used for the quantum link. The crosstalk noise is reduced using a narrow spectral filter at the Hub.

Hub is capable of switching between different spokes by default in a round-robin manner and on-demand request by any spoke depending upon the secure key requirement. For this, quantum nodes in ChaQra maintain a key store with respect to the physically linked direct quantum peer. The connected node also provide the health status to trigger retuning process on a system restart. Hub performs clock synchronisation and offset calculation with the spokes at the first start and re-perform it if required.

### Architecture

ChaQra is a three-layer architecture based on the key relay via Hub to perform key migration between any two spokes. The quantum layer consists of point-to-point connection between a single spoke and the Hub. Each such point-to-point connection generates their secure keys using the (Decoy) Differential Phase Shift (DPS) protocol during its connection period. The protocol selection is based on the link loss (DPS till 20 dB and Decoy-DPS till 33dB). The generated keys (1024 bits) are stored in their key store by assigning a key-ID to each key. The key store is a quantum vault where stored keys are secured by a master key. This key store is referred as the key management layer. A key is retrieved by an API (Application Programming Interface) call to the assigned key-ID. The software compatibility of the common API provides the interoperability of different QKD devices.

Each spoke device has a key management agent (KMA) whose job is to resize the key materials for absorbing the difference in key generation rate and key length of each QKD link, to reshape the key materials into a common format for further use, and to supply unique identifiers to the key materials. It arranges the materials sequentially to synchronize key usage for encryption and decryption. Additionally, it records statistical data like quantum bit error rate (QBER) and key generation rate. These data are then forwarded to the centralized key network management station located at the Hub. The Hub coordinates and oversees all links in the network and has key management server (KMS). Networking operations are exclusively handled within the KM layer by software under the supervision of the KMS. Spokes can use KMA to relay a secure key from one node to a second node by OTP-encrypting the key, using another key shared with the second node using Hub as a relay trusted node. Thus a secure key can be shared between different spokes that are not directly connected to each other by a quantum link. The KMS manages the key life cycle. The authentication is done by the Wegman-Carter scheme, based on using previously-generated secure key. The respective code is implemented in the KMS and KMAs. All the KMAs are located inside the network isolated from each other by L2 switches. The network itself is connected to the Internet via a router. The third layer (Application layer) fetches the key using either ETSI based key interface or north bound interface.

In the Application layer, secure communication is guaranteed through the utilization of distributed keys for encrypting and decrypting text, audio, or video data generated by diverse applications. Users reside within the trusted nodes. User data are sent to KMAs, and encrypted and decrypted by OTP in a stored key mode. Besides OTP encryption, Advanced Encryption Standard (AES-256) is also implemented in each KMA. The KMS switches two cryptographic schemes, referring to residual amounts of secure keys. Any spoke can request a connection to the Hub for key generation depending upon the residual secure keys and the foreseen key requirements.

### Software defined network (SDN)

The current academic research on QKD concentrate, almost exclusively, on maximizing the operation distance or key throughput. To achieve this, their architecture has been tuned to minimize any disturbance in the quantum channel. Thus, the use of dark fiber for the quantum channel has been prevalent. In fact, most of these networks can be seen as an ad-hoc, separate network, built solely for quantum purposes that use any classical network available for the associated classical communications only. This approach requires to build a specific infrastructure just for QKD. While this might be adequate for early adopters or research motivated but temporally QKD testbeds, it presents several challenges for its wide-spread usage. Not reusing or not sharing existing infrastructure is very costly and demands a large investment up-front. It is not only about optical fiber, but also about additional management costs and sub optimal use of the network, dealing with proprietary interfaces, specialized maintenance, and, in general, a lack of flexibility and interoperability. Such designs inhibit a scale-up of the network and adding systems in a multi vendor infrastructure.

**Figure 2:**
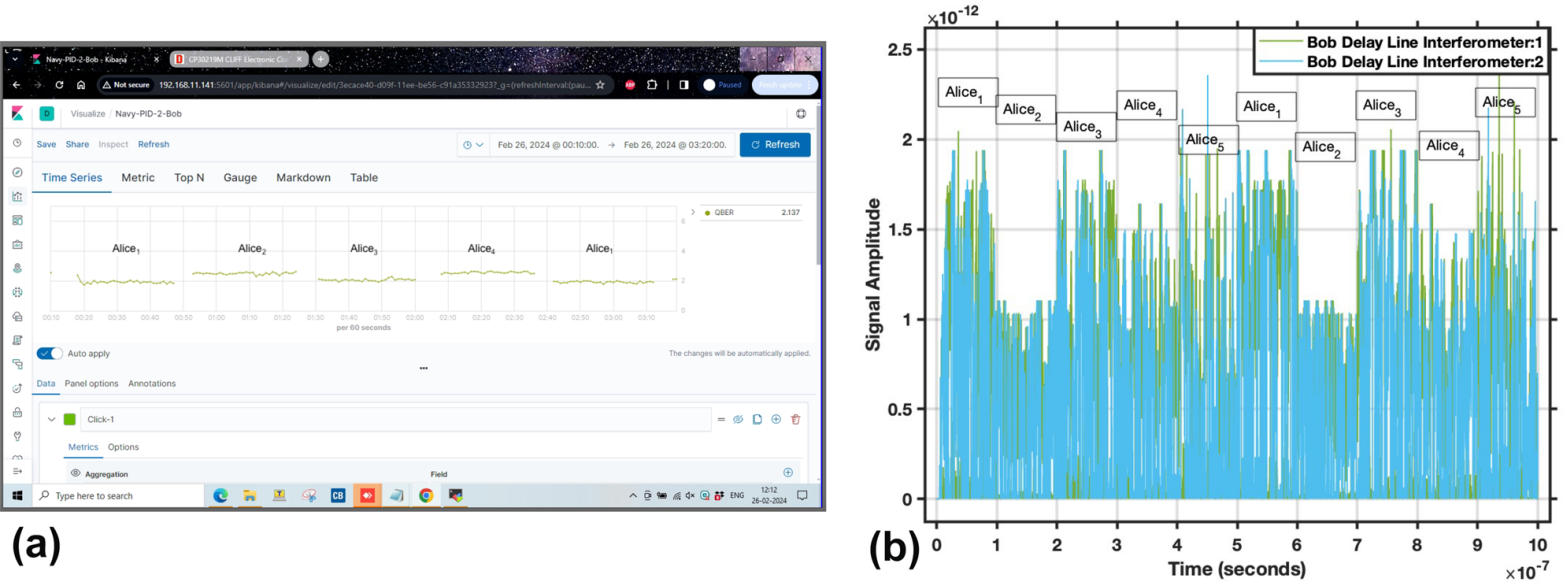
Dynamical switching mechanism in ChaQra (**a**) setup and in ChaQra simulation environment (**b**). (**a**) Dynamical switching of the spokes using the centralised SDN at Hub with the switching time less than 2 minutes at the first round. Quantum bit error rate (QBER) is less than 2% and 3% for Alice (1, 3) and (2, 4) respectively just after the switching time as well. (**b**) Dynamical switching of the spokes in the round-robin fashion in the experimental simulation environment of the ChaQra.

To avoid these problems, the network presented here was built following a completely different approach. Its architecture follows the SDN paradigm, designed to increase the flexibility and shorten the times for deployment and maintenance. Standards and well-known tools in the telecommunications industry were extensively used to facilitate integration and adoption. The fundamental concept of SDN is the separation between the control and data planes. In an SDN environment, the data plane, considered as the set of data and functionalities provided by the network to ensure traffic from source to destination, is bounded to dedicated elements (forwarding functions or devices). In an SDN based QKD network, QKD nodes provide programmable NBI (North Bound Interface) to allow switching of the key generation process to other QKD node physically attached in the ChaQra network as shown in Fig. 2. The average switching time from one spoke to another is less than 2 minutes (Fig. 2a). We have simulated the switching mechanism in our Matlab simulator as well (Fig. 2b). An application interface is provided based on ETSI GS QKD $004-v2.1.1$. A CONNECT interface for any application to reserve an association (Keystream ID) for a set of future keys. It return Keystream ID on successful establishment of the key path. Keystream ID is defined by the applications and provided to the CONNECT interface as an input. It allow the application to define the spoke with which the Key path has to be established. It allow the application to define the Quality of Service according to the following parameters—key chunk size, Max and Min bps, jitter, priority, timeout, time to live (TTL). It also provide the application to subscribe for a specified key chunk size in bytes. SDN also has a CLOSE interface for an application to terminate the established Keystream ID. SDN provide a GETKEY interface allowing for the client/server of the application to get a key of the chunk size defined in the CONNECT interface. GETKEY interface returns an index parameter specifying the position of the key to be accessed within the reserved key store for the application.

A centralized network monitoring (generation key rate, node availability, available key count) and configuration station provide control over Quality of Service (QoS) requirements of different applications (ETSI GS QKD 015*v*1.1.1). The centralised node caters to the on-demand connection requests of the spoke nodes. It provide reconfiguration of the key relay path in any failure scenario (ETSI GS QKD 015*v*1.1.1). It also provide an optimum key relay path for a virtual link between any two spokes. Each QKD node provides a QKDID (QKD interface ID) which is locally unique and when combined with SDN-QKD nodes ID becomes globally unique. Each QKD node provides the QKD role it can support TRANSMITTER, RECEIVER or BOTH (ETSI GS QKD 015*v*1.1.1). Each QKD node provides the maximum channel loss it can support (dB) which is a function of the QKD protocol it can perform. Each QKD node provides a unique ID of the optical switch to which the QKD interface is connected. Key Management layer shall send a QKD-SDN application new notification to the SDN controller upon receiving a request from an application to open a new connection for key fetching. SDN controller reserves a bandwidth from the intermediate nodes to support the virtual key association link as an internal application. We next discuss the network extension capabilities of the QKD nodes.

### Network extension

**Figure 3:**
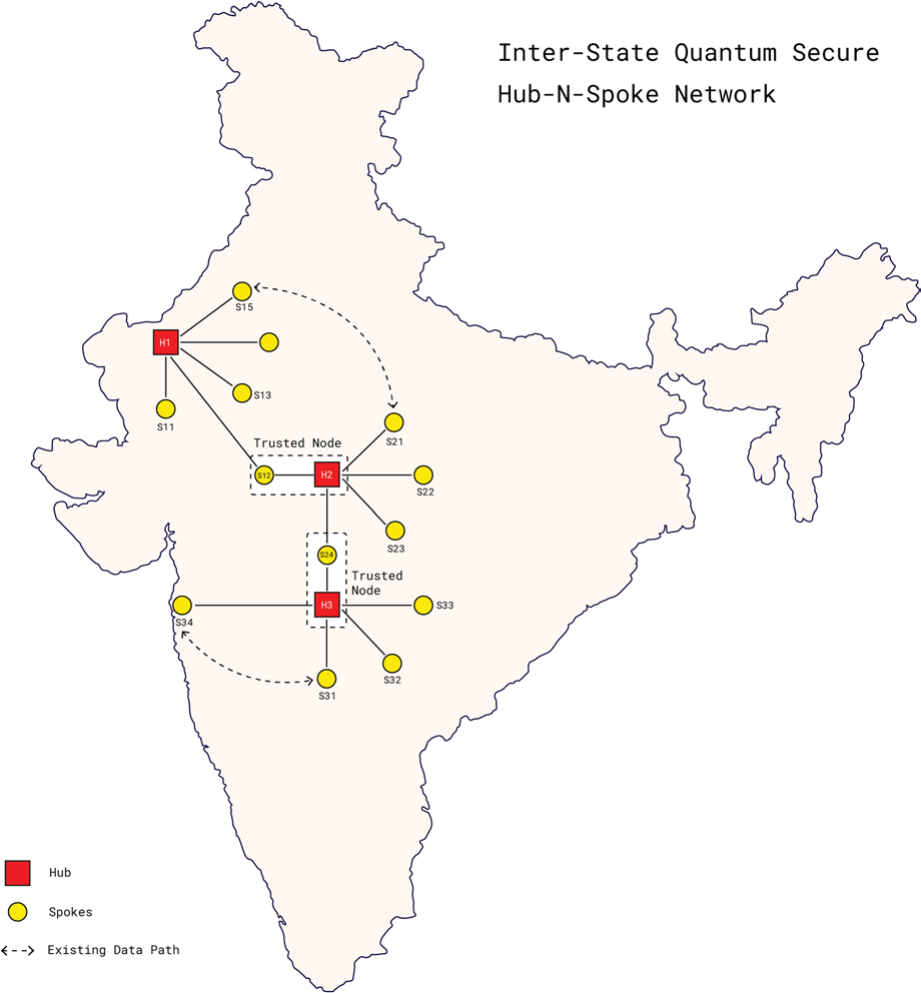
Network extension to the Indian subcontinent using ChaQra as a cellular unit.

The network extension of chaQra beyond a single Hub and five spokes is made possible with the help of a trusted intermediate node. Each QKD node is capable to function either as an end node or a trusted relay. The approach is scalable and flexible: it can be extended to many nodes, it can be used to increase the capacity of each node, it supports a variety of QKD technologies and even supports new services beyond QKD. Further, there is a possibility to develop photonic integrated circuit module for the Spoke node in near future and a combined transceiver module as the network scales. Figure 3 highlights the network extension approach across Indian subcontinent. The components within a node are:

- *Local Key Management System (LKMS):* it collects the keys from the QKD modules and serves the applications; indexes and stores the generated keys, manages their life cycle, and keeps track of the key-generation peers; provides information on key availability to the SDN controller through the SDN agent and the keys to be forwarded when needed. Each trusted region’s key management layer maintain a key store based on the virtual links it is part of as an originator or destination. Below we use the general term Key Management System (KMS) to address the functionalities of the set of all LKMSs.
- *Forwarding Module:* It is in charge of the key transport between nodes using the shared keys created by the QKD module pairs. In contrast to typical implementations, this functionality is here separated from the LKMS, since key routing is not a part of KMS duties as defined in, e.g., the NIST SP 800 document series. This facilitates the integration into the standard security ecosystem.
- *SDN Agent:* SDN controller counterpart in each node that connects the controller with all the components within the node. Note that security-sensitive information is not available to the control mechanism.
- *QKD Module:* The quantum sender/receiver itself, which continuously generates the keys. In general, there are three channels associated to it: the quantum channel, a service channel, needed to stabilize the quantum channel (possibly integrated with the former), and the classical key-distillation channel.
- *Application:* Any entity, inside the SDNQKD security perimeter, requesting QKD keys from the LKMS. The applications might be external, e.g., an end-user application, a hardware security module (HSM), a virtual network function, or internal with respect to the key distribution functionality, e.g., authentication, virtual link management, or key transport. The applications use the application interface implemented in the LKMS to obtain the key material.

### Q-infinity (addressing denial-of-service)

Although QKD offers the theoretical security, leveraging the uncertainty principle and no-cloning theorem of quantum mechanics, it has some major challenges (limited distance, denial-of-service, end user connectivity) to overcome to achieve its full potential and scalability. Here, denial-of-service is not confined only to the scenario of eavesdropping but also to the channel disruption.

QNu Labs Q-infinity addresses the denial-of-service in such situations by deriving keys to the authenticated nodes using a hybrid approach of quantum safe cryptography during the period when QKD link is not operational. It derives the symmetric keys using hybrid approach incorporating multi layers (Post Quantum Cryptography + steganography + Galois field) for post-quantum security of 401 bits. Q-infinity makes use of the QKD standard ETSI-014 interfaces.

Next we describe the two categories of the QKD systems constituting ChaQra.

## QKD systems

**Figure 4:**
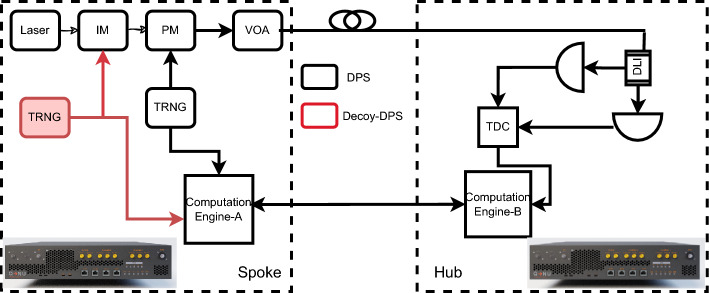
Block diagram of the QKD systems constituting ChaQra. Armos (DPS) is a version supporting secure distance upto 100 km (20 dB) and Armos 2.0 (Decoy-DPS) supports the secure distance upto 150 km (33 dB) with the standard single photon detectors. Note that ChaQra contains a single Hub (QKD receiver) compatible with both the QKD systems.

The quantum key distribution protocol used by the spokes to establish a secure key with the Hub is Differential phase shift (DPS) upto 100 km (20 dB) or Decoy-DPS upto 150 km (33 dB) as shown in Fig. 4. A continuous wave laser at the spoke node is modulated into a train of pulses with an intensity modulator (IM) at the repetition rate of 1 Gbps. These pulses then pass through a phase modulator where a random phase of 0 or $\pi$ is given to each pulse depending upon the outcome of the true random number generator (TRNG). These pulses are then attenuated to the single photon regime using the variable optical attenuator (VoA). The encoded weak coherent state (encoding in the phase degree of freedom) propagates through the fiber quantum channel to the Hub node, where it enters a one-bit-delay Mach Zehnder Interferometer (MZI) for the demodulation. Inside the one-bit delay MZI (DLI), each pulse splits at the first beam splitter and interferes with its neighboring (next) pulse at the second beam splitter. Depending upon the phase difference of the neighbouring pulses either the SPD-1 (0 phase difference) connected at the channel-1 of the DLI gets a detection or the SPD-2 ($\pi$ phase difference) connected at the channel-2 of the DLI gets a detection.

The mean photon number (MPN) of the one kind of pulses is kept below 0.1 in the DPS protocol. In the Decoy-DPS protocol, there are three kind of pulses: 1. Signal pulses (MPN $\le$ 0.7), Decoy (MPN $\le$ 0.2), and Vacuum (MPN $\le$ 0.0001). These pulses are generated using the IM by utilizing the range of its transfer curve. There is no characteristic difference between the three kind of pulses except the MPN.

In this protocol, the increased mean -photon-number (MPN) of the signal state leads to higher key distribution rates and greater operational distances. This is because the percentage of single photon pulses increases till MPN of 1 for a light source following the Poissonian distribution. Also, utilizing different MPNs for the signal and decoy states, allows the QKD system to detect photon number specific interference using the decoy state protocol security condition. The outline of the Decoy-DPS (DDPS) protocol is as follows:

- *Quantum Information Exchange* It involves generation, transmission and detection of decoy and signal photon pulses by the end users. Both kind of pulses have identical characteristics except the MPN. Only Alice node knows which pulse is signal/decoy throughout the quantum information exchange stage. At the end of this stage, Bob shares the time stamp data of the detection events to Alice.
- *First stage of security check* Alice uses the time stamp information from Bob node to determine photon dependent yield ($y_n$) for decoy and signal pulses. At this point, Alice can ABORT/ CONTINUE the secret key generation depending upon the baseline yield of decoy and signal pulses and their tolerance value ($10^{-5}$).
- *Post-processing* Alice discard all the pulses which are not detected by Bob. During bi-directional error correction procedure, Alice asks bit values corresponding to all the decoy pulses and 15 $\%$ of the randomly chosen signal pulses from Bob. Alice randomly selects the time stamp corresponding to signal or decoy pulse and asks whether that corresponds to Bob’s detector-1 or detector-2. This way, she need not to disclose the identity of the pulses. She then estimate the photon dependent QBER ($Q_n$) of decoy and signal pulses and their tolerance value.
- *Second stage of security check* If the QBER statistics also matches, then Alice finally proceeds for the error correction for the remaining signal clicks and applies privacy amplification.
- *Secret key generation* Alice calculates the lower bound of the single photon yield and upper bound on the single photon QBER and calculates the theoretical secret key rate. She uses this to determine the compression to be applied on the error corrected raw secret keys. Alice and Bob then compress the keys and generate the secure keys. We conducted the statistical randomness testing of the generated keys and all tests passed.

After the execution of the key generation protocol, QKD nodes have the following characteristic parameters as follows (Table 2).

**Table 2:** Key specifications of ChaQra. Key rate at lesser loss is limited by the dead time of the single photon detector.

S. no.	Spoke no.	Distance (km)	Loss (dB)	Key rate (kbps)	QBER (%)
1.	A1	100	28	3.2	3.66
2.	A2	90	25	6.4	3.34
3.	A3	75	18	9.8	3.02
4.	A4	65	15	16.2	2.34
1.	A5	100	30	1.8	3.5

## ChaQra: experimental simulation

**Figure 5:**
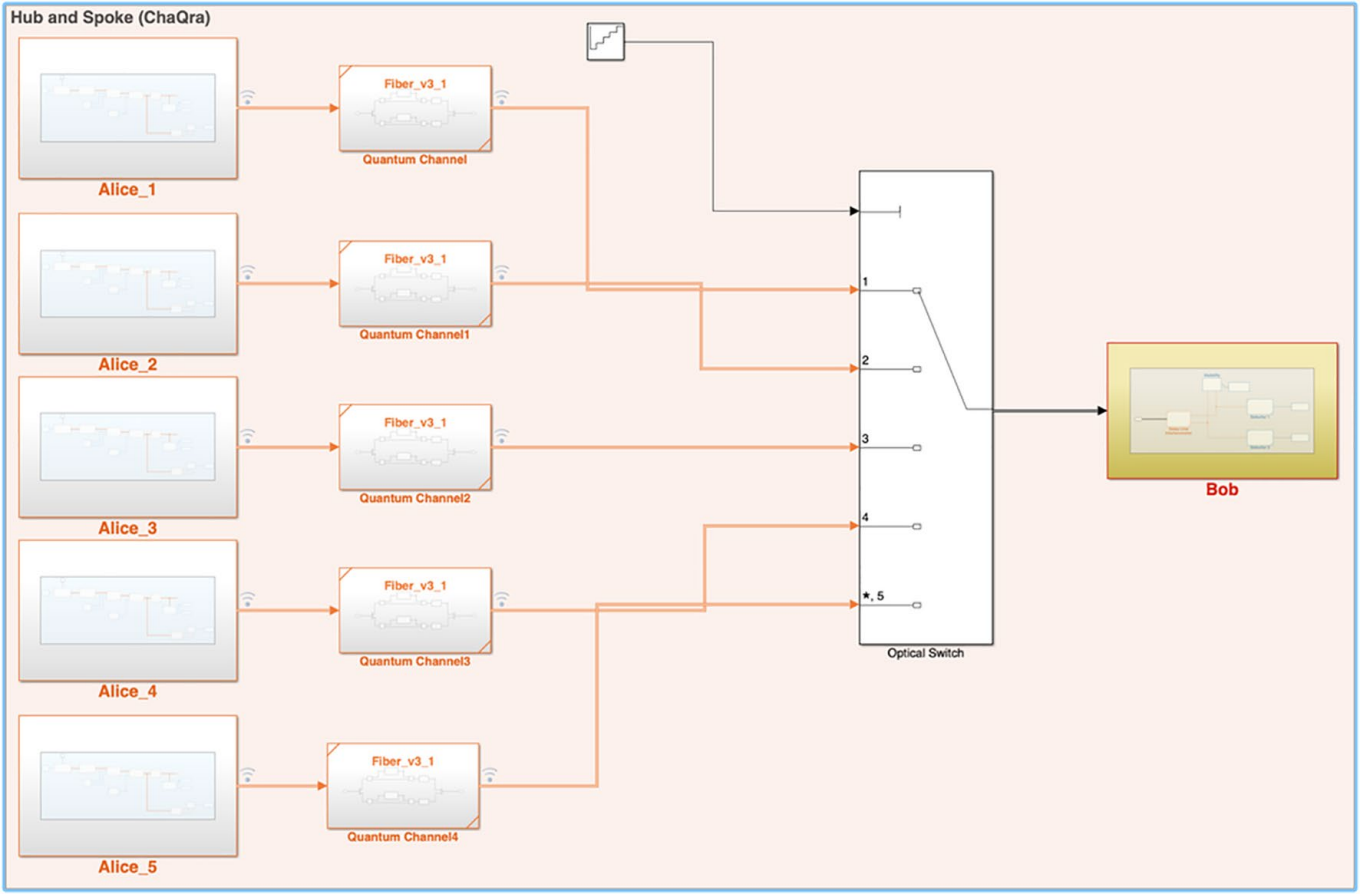
Simulink block diagram of ChaQra.

In this section, we describe the modelling scheme for each module representing a physical device (QKD node and its constituting elements). Similar to physical system, the model has Hub, quantum channel, optical switch and Spoke subsystem as shown in Fig. 5. We first discuss the Hub node constituting the optical switch followed by the Spoke node.

### Hub node

**Figure 6:**
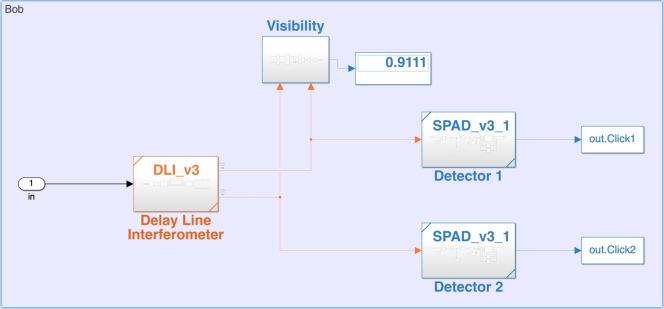
Simulink block diagram of the Hub node.

Hub module simulates the detection of the signal photons transmitted by Spokes over the quantum channel modelled by the optical fiber to estimate the photon dependent yield of the signal and decoy pulses and their QBER value. Hub detection module is constructed using the sub-modules like beam splitters, delay-line interferometer, and single- photon detectors. All these components together are used to perform a Mach-Zehnder interference between neighbouring pulses. This produces photon clicks at either of the two detectors based upon the phase difference between the neighbouring pulses. The block diagram is shown in the Fig. 6. The switching mechanism of the Hub node is controlled using the optical switch module as follows:

#### Optical switch module

The optical switch module in the Simulink is designed using a multilevel switch constituting five input ports and single output port. The selection of the input port is based on the repeating sequence which is by default a round-robin pattern. Depending upon the repeating sequence value at a given time, a specific spoke node establishes a connection with the Hub for the key generation. The switching of the spoke nodes is captured in the Fig. 2b.

### Spoke node

**Figure 7:**
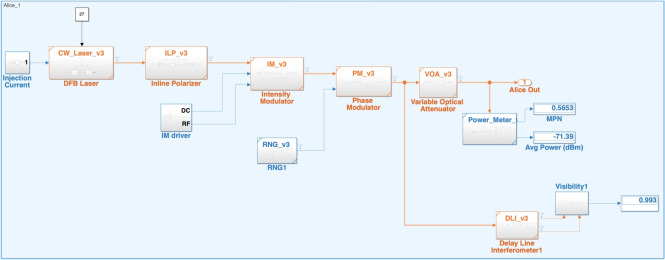
Simulink block diagram of Spoke node.

Spoke node in ChaQra comprises of a laser source and a quantum state preparation components. In practise, the source is a laser which is attenuated to the quantum level (MPN < 1). This results in a coherent state with MPN less then unity, known as weak coherent state. This weak coherent source is then subject to phase modulator. The block diagram for the simulation can be seen in Fig. 7. The detailed component level simulation and signal propagation are provided in the paper^41^. The objective of the Alice node is to create decoy (MPN $\le$ 0.2) and signal (MPN $\le$ 0.7) weak coherent pulses randomly encoded in the 0 or $\pi$ phases and to keep a record of the XOR value of the phase of the prepared pulses with the phase of the one-bit shifted pulses. The QKD protocol is continued only if the QBER is within the threshold (< 4%). The selection of the spoke for the key generation is based on the repeating sequence pattern.

## Methods

In this section, we elaborate different layers and interfaces to constituting ChaQra.

### Quantumness and structured unit

Each QKD node in ChaQra sub-network consists of a 2U rack mountable unit. In order to optimise the key specification like key rate, error rate and security there is a provision to switch to decoy based DPS protocol from traditional DPS protocol. The error correction process utilizes a multi threaded version of the Cascade protocol. To minimize the effects of finite key size, we use large sifted block sizes of 8 MB. The choice of 8 MB for the sifted block size is based on the trade-off between the efficiency and speed of the privacy amplification algorithm. Post-processing of 8 MB of the sifted data is completed in a cycle of 68 milliseconds. Privacy amplification is implemented using a number-theoretic transform method in an FPGA^52^. The security parameter for privacy amplification was chosen to be $9 \times 10^{-13}$. The overall security parameter estimated is $\epsilon _{\text {Armos}} \equiv 3 \times 10^{-12}$. The detection efficiency is around $10\%$ to keep jitter and after-pulsing effects low, and it can go up to $30\%$.

For the quantum key distribution (QKD) classical channels, they are wavelength-multiplexed onto the same fiber as the quantum channel and consist of synchronization and reconciliation channels (channel-34). Note that the reconciliation channels include a standard bi-directional 1G Ethernet link. Raman scattering noise and cross-talk from the subsequent channel are minimized using a narrow spectral filter at the entrance of the Hub node (channel-37).

### Secure key delivery module

The secure key delivery module, found at each node in the ChaQra sub-network, includes four sub-modules: quantum key generation, OTP based key relay, global key management, key migration and an API. The module is efficiently implemented in software to handle high quantum key rates. Quantum keys are pushed from QKD systems interface to the key delivery modules via a secure link and buffered locally. Global keys are locally generated and securely routed to peer nodes via OTP encryption of quantum keys. For secure key relaying and migration, the next node is determined by a routing table lookup at the intermediate node. The global key management sub-module tracks the number of stored global keys and calls the OTP key relay sub-module to replenish them as needed. Multiple users connect to the secure key delivery module to request global keys for their applications using a REST-style API, which uses HTTPS and JSON. Users are authenticated through a certificate-based scheme, passcode, and fingerprint. The HTTPS and JSON protocols are used only within the trusted node between the key management and application devices^52^.

### Network extension using trusted-nodes

At present, trusted nodes are a common way to extend QKD networks beyond metro area networks, like the one connecting Beijing and Shanghai in China. These networks have traditionally relied on trusted nodes, but now there’s growing interest in building QKD networks using untrusted relays or quantum repeaters. However, this is still at a primitive stage and far from deployment in the existing optical infrastructures. Even though it’s impossible to have a completely secure node, in the real world, many nodes in realistic settings are very secure like a data centre. They often need high-level permission to access, and the network equipment is kept in tamper-proof containers to prevent unauthorized meddling. Therefore, it is the best option at present for the QKD network extension that provides quantum security at the optical links and best possible classical security at the intermediate nodes.

### Beyond QKD

The private randomness generated in the ChaQra sub-network enables complex tasks that require collaboration and privacy among multiple parties. For example, it allow different organizations to jointly perform data analysis or decision making without revealing their individual inputs. This can enhance the efficiency, security and trustworthiness of the network and a great value addition^40^. As an example, let us consider that the five spokes wants to compute the sum of their private inputs. The objective is to compute the sum keeping their inputs secrets ($a_1, a_2, a_3, a_4, a_5$). This is typical multiparty computation problem that is traditionally been solved using classical cryptographic primitives like secret sharing or oblivious transfer. However, in a QKD network, the spokes can leverage the QKD keys to compute the sum or any other multiparty computation problem as follows:

- *Step-1.* Let the shared QKD keys between Alice$_1$ and Alice$_2$, Alice$_2$ and Alice$_3$, Alice$_3$ and Alice$_4$, Alice$_4$ and Alice$_5$, and Alice$_5$ and Alice$_1$ are $X_{1,2}$, $X_{2,3}$, $X_{3,4}$, $X_{4,5}$, and $X_{5,1}$ respectively.
- *Step-2.* Alice$_1$ computes $A_1 = a_1 + X_{1,2} - X_{5,1}$ which is random. Similarly, Alice$_2$, Alice$_3$, Alice$_4$, Alice$_5$, computes $A_2 = a_2 + X_{2,3} - X_{1,2}$, $A_3 = a_3 + X_{3,4} - X_{2,3}$, $A_4 = a_4 + X_{4,5} - X_{3,4}$, $A_5 = a_5 + X_{5,1} - X_{4,5}$ respectively. $A_1, A_2, A_3, A_4, A_5$ being random are publicly announced by the spokes. Note that the Hub is the trusted node in our setup.
- *Step-3.* The sum (S) = $A_1 + A_2 + A_3 + A_4 + A_5 = a_1 + a_2 + a_3 + a_4 + a_5$. The privacy of the inputs is ensured by the QKD keys derived using the ChaQra.

## Discussion

We have demonstrated ChaQra—a cellular unit of the Indian Quantum Network. In this network, five QKD transmitter node named as spokes establishes connection with a single QKD receiver node (Hub) in a time-division multiplexing. They were interconnected via several key management agents using a common API, and managed by a single key management server. The demonstrated applications include secure video-conferencing (polycom). The former application was supported between the two spokes node, each of which are connected using the Hub as a trusted relay node, and by a rerouting function to switch from a hacked to an alternative secure link (Q-infinity) when an eavesdropper in the system was detected or during the other denial-of-service instances. These demonstrations suggest a clear picture for a wider quantum secure network in the context of Indian sub continent to secure the critical infrastructure in the post-quantum era.

The near-term use case is likely to be high-end security applications of dealing with national security that have been relying up to now on vulnerable classical cryptography. To become a practical solution long-term reliability of QKD needs to be guaranteed. This includes not only stable operation but also security assurance over a long span of time. In this regard, an active stabilization schemes and as many side-channel countermeasures as possible without sacrificing performance should be continuously undertaken. At present, we deployed circulators, narrow band spectral filters and isolators at appropriate places to with stand major side-channel attacks. In the current version, we have addressed some of the issues highlighted in the other QKD network across the globe such as integration of the QKD link in the already deployed telecommunication fibres. We have developed a sophisticated Software defined networking based on the guidelines of ETSI to create a cost-effective, scalable P2M sub-network. We have also addressed denial-of-service with hybrid quantum safe cryptography that can be more efficiently handled when we have a more complex network containing redundant QKD links. Further, we propose over network to be beyond QKD that can be used for distributed computing, multi-party computation adding more value to the network. Finally, we have adopted a modular architecture to upgrade the network with the scientific and technological development in the long run. We have a provision to look for multi-protocols support and look for other important quantum communication tasks like quantum secure direct communication, quantum secret sharing as a part of ChaQra in the future.

## Data Availability

The datasets generated during and/or analyzed during the current study have are available from the corresponding author on reasonable request (https://github.com/shashankg687/ChaQra).
